# Supplementary material for: Higher Intake of Fat, Vitamin E-(β+γ), Magnesium, Sodium, and Copper Increases the Susceptibility to Prostatitis-like Symptoms: Evidence from a Chinese Adult Cohort
Source: Nutrients. 2022 Sep 6;14(18):3675. doi: 10.3390/nu14183675 (PMC9501331; doi:10.3390/nu14183675)
Supplement: Supplementary file 1 [file nutrients-14-03675-s001.zip › nutrients-1876545-supplementary/nutrients-1876545- supplementary.pdf]

Excessive Intake of Fat, Vitamin E-(β+γ), Magnesium, Sodium, and Copper Increase the Susceptibility to Prostatitis-like Symptoms: Evidence from a Chinese Adult Cohort

Meng Zhang[1,2,3#], Chen Jin[1,2,3#], Yang Ding[4#], Yuqing Tao[4], Yulin Zhang[4], Ziyue Fu[4], Tao Zhou[5], Li Zhang[1,2,3], Zhengyao Song[1,2,3], Zongyao Hao[1,2,3], Jialin Meng[1,2,3]*, Chaozhao Liang[1,2,3]*

[1]Department of Urology, The First Affiliated Hospital of Anhui Medical University, Hefei, 230022, P.R. China;

[2]Institute of Urology, Anhui Medical University, Hefei, 230022, P.R. China;

[3]Anhui Province Key Laboratory of Genitourinary Diseases, Anhui Medical University, Hefei, 230022, P.R. China;

[4]The Second Clinical Medical College, Anhui Medical University, Hefei, 230022, P.R. China;

[5]The First Affiliated Hospital of Anhui Medical University, Hefei, 230022, P.R. China.

[#]These authors contributed equally to the study.

*__Correspondence to:__

Jialin Meng Ph.D. & M.D.

Email: mengjialin@ahmu.edu.cn

Chaozhao Liang Ph.D. & M.D.

Email: liang_chaozhao@ahmu.edu.cn

Tel./Fax: +86 55162922034

Address: Jixi Road 218, Shushan District, Hefei City 230022, Anhui Province, People's Republic of China.

ORCID to:

Jialin Meng: 0000-0002-4622-833X

Chao Zhao Liang: 0000-0003-2317-1323

Meng Zhang: 0000-0003-4935-4005

Chen Jin: 0000-0001-6803-5785

Running title：Dietary predisposing factor for prostatitis-like symptoms

Supplementary Table S1. Dietary questionnaire used to collect data in current study.

A. Diet questionnaire

1. Milk and products

What kind of food is involved in the daily diet: milk () milk powder () yogurt () cheese () The above are not often involved()

2. Beans

Which of the following foods are involved in daily diet: tofu () bean curd cake () thin sheets of bean curd () dried bean curd stick () soybean () green bean () kidney bean () eyebrow bean () pea () soya-bean milk () The above are not often involved ()

3. Fish, shrimp, and shellfish

Which of the following foods are involved in daily diet: fish () shrimp () dried shrimp () river crab () swimming crab () screw () The above are not often involved ()

4. Livestock, poultry, meat, and eggs

Which of the following foods are involved in the daily diet: pork () trotter () rib () beef jerky () beef () beef haslet () mutton () sheep haslet () chicken () chicken breast () chicken leg () chicken feet () chicken gizzards () chicken wing () duck meat () goose meat () egg () scrambled and fried egg () The above are not often involved ()

5. Cereals

Three main meals on weekdays include: rice () fine dried noodle () fresh noodle () steamed bread () bread () porridge () millet porridge () fried dough sticks () baked wheat cake () oatmeal () The above are not often involved ()

6. Vegetables

Daily vegetables mainly include: nori (dry) () edamame () rape () amaranth () celery () nappa cabbage () pakchoi () lettuce () spinach () broccoli () anglin (cabbage) () kelp () leek () lotus root () garlic () onion () carrot () eggplant () bean (cowpea) () cucumber () black fungus (dry) () asparagus lettuce () soybean sprout () zucchini () tomato () cauliflower () The above are not often involved ()

7. Nuts

Does the daily diet involve the following foods: peanut () watermelon seed () sunflower

seed () sunflower seed kernel () pumpkin seed () chestnuts () pine nut () jujube () lotus seed () walnut () The above are not often involved ()
**8. Fruit**
Which of the following foods are involved in the daily diet: apple () banana () peach () kiwi () mulberry () raisin () watermelon () pear () citrus () crabapple (hawthorn) () The above are not often involved ()

Note: Dietary records were made based on the retrospective dietary survey chart[1], and the Chinese food composition tables[2] were used to assist in estimating the average daily element intake.

Reference

1. Ding Y, Yang Y, Li F, Shao Y, Sun Z, Zhong C, Fan P, Li Z, Zhang M, Li X, Jiang T, Song C, Chen D, Peng X, Yin L, She Y, Wang Z (2021) Development and validation of a photographic atlas of food portions for accurate quantification of dietary intakes in China. J Hum Nutr Diet 34(3):604-615. http://doi.10.1111/jhn.12844
2. Yang Y, Wang Z, He M, Pan X. (2019) Chinese Food Composition Table Standard Edition. 6th edn. Peking University Medical Press, Beijing China

Supplementary Table S3. Multiple logistic regression models were constructed by adding confounding factors gradually to identify high-risk elements intake associated with PLS in daily diet (continued).

		Model 1		Model 2		Model 3		Model 4		Model 5	
Variables	No.	OR (95% CI)	*P*-value	OR (95% CI)	*P*-value	OR (95% CI)	*P*-value	OR (95% CI)	*P*-value	OR (95% CI)	*P*-value
Moisture (g)											
IQR1	172	Ref.		Ref.		Ref.		Ref.		Ref.	
IQR2	166	0.968 (0.609,1.538)	0.890	1.007 (0.626,1.620)	0.977	0.989 (0.614,1.595)	0.965	1.005 (0.612,1.652)	0.984	1.000 (0.608,1.645)	0.999
IQR3	146	1.318 (0.827,2.101)	0.246	1.231 (0.760,1.995)	0.398	1.215 (0.748,1.973)	0.431	1.253 (0.721,2.176)	0.424	1.219 (0.696,2.134)	0.488
IQR4	164	1.262 (0.801,1.987)	0.316	1.146 (0.716,1.834)	0.571	1.172 (0.731,1.880)	0.510	1.256 (0.586,2.695)	0.558	1.204 (0.554,2.617)	0.639
Protein (g)											
IQR1	174	Ref.		Ref.		Ref.		Ref.		Ref.	
IQR2	155	1.325 (0.821,2.140)	0.250	1.186 (0.725,1.940)	0.498	1.192 (0.728,1.953)	0.485	1.222 (0.738,2.026)	0.436	1.262 (0.759,2.097)	0.370
IQR3	168	1.949 (1.233,3.083)	**0.004**	1.655 (1.033,2.651)	**0.036**	1.598 (0.994,2.568)	0.053	1.681 (0.998,2.832)	0.051	1.817 (1.061,3.110)	**0.029**
IQR4	151	1.596 (0.992,2.567)	0.054	1.326 (0.812,2.166)	0.26	1.330 (0.812,2.179)	0.258	1.498 (0.738,3.041)	0.263	1.816 (0.840,3.923)	0.129
Carbohydrate (g)											
IQR1	162	Ref.		Ref.		Ref.		Ref.		Ref.	
IQR2	165	1.166 (0.722,1.884)	0.530	1.134 (0.694,1.853)	0.615	1.111 (0.679,1.820)	0.675	1.124 (0.679,1.861)	0.649	1.151 (0.694,1.909)	0.587
IQR3	159	1.760 (1.100,2.816)	**0.018**	1.575 (0.972,2.553)	0.065	1.528 (0.940,2.481)	0.087	1.565 (0.922,2.656)	0.097	1.674 (0.973,2.880)	0.063
IQR4	162	1.536 (0.959,2.461)	0.074	1.273 (0.781,2.077)	0.333	1.242 (0.761,2.029)	0.386	1.305 (0.679,2.509)	0.425	1.569 (0.759,3.243)	0.224
Dietary fiber (g)											
IQR1	159	Ref.		Ref.		Ref.		Ref.		Ref.	
IQR2	162	1.582 (0.958,2.613)	0.073	1.349 (0.808,2.254)	0.252	1.331 (0.795,2.231)	0.277	1.378 (0.816,2.330)	0.231	1.400 (0.828,2.368)	0.209

IQR3	168	2.593 (1.598,4.208)	**<0.001**	2.134 (1.300,3.505)	**0.003**	2.142 (1.302,3.526)	**0.003**	2.302 (1.347,3.935)	**0.002**	2.379 (1.386,4.082)	**0.002**
IQR4	159	2.147 (1.311,3.517)	**0.002**	1.518 (0.903,2.553)	0.115	1.538 (0.912,2.592)	0.106	1.785 (0.921,3.462)	0.086	1.936 (0.982,3.814)	0.056
Cholesterol (mg)											
IQR1	166	Ref.		Ref.		Ref.		Ref.		Ref.	
IQR2	167	1.021 (0.635,1.643)	0.931	0.904 (0.554,1.475)	0.687	0.934 (0.571,1.530)	0.787	0.957 (0.583,1.571)	0.863	0.967 (0.589,1.589)	0.895
IQR3	164	1.349 (0.846,2.150)	0.208	1.133 (0.699,1.835)	0.613	1.164 (0.715,1.893)	0.541	1.220 (0.743,2.003)	0.432	1.223 (0.745,2.008)	0.427
IQR4	151	1.863 (1.167,2.971)	**0.009**	1.591 (0.984,2.573)	0.058	1.701 (1.045,2.767)	**0.033**	1.950 (1.114,3.412)	**0.019**	1.948 (1.113,3.409)	**0.020**
Ash (g)											
IQR1	170	Ref.		Ref.		Ref.		Ref.		Ref.	
IQR2	160	1.345 (0.829,2.183)	0.230	1.165 (0.708,1.918)	0.548	1.174 (0.712,1.936)	0.530	1.218 (0.729,2.037)	0.451	1.242 (0.742,2.081)	0.409
IQR3	156	2.235 (1.395,3.581)	**0.001**	1.994 (1.232,3.229)	**0.005**	1.968 (1.212,3.198)	**0.006**	2.124 (1.237,3.646)	**0.006**	2.236 (1.289,3.879)	**0.004**
IQR4	162	1.746 (1.088,2.802)	**0.021**	1.343 (0.820,2.201)	0.242	1.344 (0.818,2.208)	0.243	1.592 (0.772,3.283)	0.208	1.853 (0.853,4.026)	0.119
Vitamin A (ug)											
IQR1	166	Ref.		Ref.		Ref.		Ref.		Ref.	
IQR2	164	2.053 (1.282,3.288)	**0.003**	1.878 (1.160,3.040)	**0.010**	1.871 (1.153,3.037)	**0.011**	1.885 (1.155,3.077)	**0.011**	1.872 (1.146,3.057)	**0.012**
IQR3	159	1.399 (0.860,2.274)	0.176	1.168 (0.706,1.933)	0.545	1.171 (0.706,1.944)	0.541	1.190 (0.701,2.021)	0.519	1.175 (0.691,1.998)	0.552
IQR4	159	1.799 (1.116,2.900)	**0.016**	1.515 (0.926,2.478)	0.098	1.579 (0.961,2.594)	0.071	1.641 (0.885,3.044)	0.116	1.609 (0.865,2.994)	0.133
Retinol (ug)											
IQR1	177	Ref.		Ref.		Ref.		Ref.		Ref.	
IQR2	173	1.062 (0.681,1.656)	0.791	1.057 (0.668,1.673)	0.812	1.094 (0.689,1.739)	0.702	1.088 (0.685,1.730)	0.72	1.074 (0.675,1.711)	0.762

IQR3	160	1.075 (0.683,1.690)	0.755	1.078 (0.677,1.717)	0.752	1.119 (0.699,1.791)	0.641	1.085 (0.666,1.770)	0.742	1.068 (0.654,1.746)	0.793
IQR4	138	0.960 (0.597,1.546)	0.868	1.006 (0.616,1.642)	0.981	1.065 (0.649,1.747)	0.803	1.006 (0.578,1.751)	0.983	1.003 (0.576,1.746)	0.991
Thiamin (mg)											
IQR1	174	Ref.		Ref.		Ref.		Ref.		Ref.	
IQR2	166	1.581 (0.993,2.516)	0.054	1.481 (0.918,2.389)	0.108	1.433 (0.886,2.316)	0.142	1.397 (0.851,2.291)	0.186	1.411 (0.858,2.319)	0.175
IQR3	145	1.911 (1.189,3.073)	**0.008**	1.863 (1.146,3.031)	**0.012**	1.815 (1.114,2.957)	**0.017**	1.732 (1.013,2.960)	**0.045**	1.765 (1.026,3.039)	**0.040**
IQR4	163	1.42 (0.887,2.274)	0.144	1.133 (0.693,1.853)	0.619	1.124 (0.686,1.842)	0.644	1.020 (0.520,2.000)	0.953	1.089 (0.524,2.266)	0.819
Riboflavin (mg)											
IQR1	179	Ref.		Ref.		Ref.		Ref.		Ref.	
IQR2	157	1.391 (0.874,2.215)	0.164	1.349 (0.837,2.175)	0.219	1.350 (0.834,2.183)	0.222	1.380 (0.844,2.258)	0.199	1.373 (0.839,2.247)	0.207
IQR3	159	1.524 (0.961,2.415)	0.073	1.419 (0.883,2.281)	0.148	1.471 (0.912,2.375)	0.114	1.542 (0.911,2.607)	0.107	1.570 (0.926,2.661)	0.094
IQR4	153	1.489 (0.935,2.372)	0.094	1.352 (0.837,2.186)	0.218	1.368 (0.842,2.222)	0.206	1.515 (0.768,2.990)	0.231	1.637 (0.813,3.297)	0.167
Vitamin C (mg)											
IQR1	163	Ref.		Ref.		Ref.		Ref.		Ref.	
IQR2	150	1.438 (0.885,2.336)	0.143	1.356 (0.824,2.231)	0.231	1.336 (0.810,2.203)	0.256	1.379 (0.831,2.289)	0.214	1.371 (0.826,2.276)	0.223
IQR3	166	1.383 (0.860,2.224)	0.181	1.228 (0.752,2.004)	0.412	1.184 (0.722,1.940)	0.504	1.260 (0.750,2.117)	0.383	1.244 (0.738,2.097)	0.412
IQR4	169	1.833 (1.151,2.919)	**0.011**	1.549 (0.957,2.507)	0.075	1.595 (0.983,2.586)	0.059	1.844 (1.005,3.381)	**0.048**	1.800 (0.973,3.330)	0.061
Vitamin E [total (mg)]											
IQR1	166	Ref.		Ref.		Ref.		Ref.		Ref.	
IQR2	156	1.575 (0.968,2.564)	0.068	1.388 (0.843,2.284)	0.198	1.378 (0.835,2.272)	0.21	1.428 (0.859,2.373)	0.17	1.445 (0.869,2.403)	0.156

IQR3	161	1.726 (1.068,2.791)	**0.026**	1.412 (0.861,2.315)	0.172	1.418 (0.862,2.332)	0.169	1.521 (0.898,2.576)	0.119	1.559 (0.919,2.645)	0.100
IQR4	165	2.154 (1.343,3.454)	**0.001**	1.645 (1.007,2.689)	**0.047**	1.660 (1.013,2.720)	**0.044**	1.911 (1.045,3.496)	**0.036**	2.147 (1.145,4.027)	**0.017**
Vitmain E-δ (mg)											
IQR1	154	Ref.		Ref.		Ref.		Ref.		Ref.	
IQR2	165	1.115 (0.691,1.798)	0.656	1.061 (0.651,1.729)	0.814	1.100 (0.673,1.797)	0.705	1.110 (0.675,1.826)	0.681	1.103 (0.670,1.814)	0.700
IQR3	171	1.148 (0.716,1.843)	0.566	0.983 (0.603,1.602)	0.945	1.000 (0.612,1.635)	0.999	1.018 (0.610,1.699)	0.946	1.022 (0.612,1.706)	0.933
IQR4	158	1.649 (1.030,2.640)	**0.037**	1.332 (0.817,2.170)	0.250	1.369 (0.836,2.240)	0.212	1.419 (0.796,2.531)	0.236	1.433 (0.803,2.558)	0.223
Calcium (mg)											
IQR1	172	Ref.		Ref.		Ref.		Ref.		Ref.	
IQR2	176	1.194 (0.761,1.873)	0.441	1.086 (0.683,1.728)	0.727	1.068 (0.669,1.703)	0.783	1.007 (0.625,1.623)	0.976	1.006 (0.625,1.621)	0.980
IQR3	151	1.399 (0.881,2.223)	0.155	1.310 (0.814,2.107)	0.266	1.324 (0.822,2.134)	0.249	1.169 (0.695,1.967)	0.555	1.167 (0.693,1.963)	0.562
IQR4	149	1.063 (0.662,1.709)	0.800	0.860 (0.524,1.412)	0.550	0.853 (0.518,1.405)	0.532	0.656 (0.338,1.273)	0.213	0.667 (0.341,1.304)	0.236
Zinc (mg)											
IQR1	171	Ref.		Ref.		Ref.		Ref.		Ref.	
IQR2	165	1.291 (0.802,2.078)	0.293	1.235 (0.758,2.012)	0.397	1.239 (0.760,2.022)	0.390	1.264 (0.765,2.087)	0.36	1.296 (0.783,2.145)	0.313
IQR3	160	2.189 (1.376,3.481)	**0.001**	1.962 (1.216,3.163)	**0.006**	1.901 (1.176,3.074)	**0.009**	1.981 (1.165,3.368)	**0.012**	2.100 (1.221,3.615)	**0.007**
IQR4	152	1.501 (0.929,2.424)	0.097	1.288 (0.784,2.115)	0.318	1.294 (0.786,2.130)	0.311	1.416 (0.702,2.856)	0.332	1.660 (0.776,3.550)	0.191
Selenium (mg)											
IQR1	165	Ref.		Ref.		Ref.		Ref.		Ref.	
IQR2	168	1.553 (0.965,2.500)	0.070	1.386 (0.852,2.257)	0.189	1.378 (0.845,2.248)	0.199	1.410 (0.858,2.319)	0.175	1.467 (0.889,2.420)	0.134

IQR3	153	1.796 (1.109,2.907)	**0.017**	1.468 (0.892,2.416)	0.131	1.436 (0.871,2.370)	0.156	1.508 (0.884,2.573)	0.132	1.622 (0.941,2.798)	0.082
IQR4	162	1.827 (1.136,2.937)	**0.013**	1.493 (0.915,2.439)	0.109	1.475 (0.899,2.419)	0.124	1.648 (0.860,3.159)	0.133	2.096 (1.009,4.354)	**0.047**
Phosphorus (mg)											
IQR1	171	Ref.		Ref.		Ref.		Ref.		Ref.	
IQR2	169	1.276 (0.799,2.037)	0.307	1.142 (0.706,1.849)	0.589	1.123 (0.692,1.823)	0.639	1.124 (0.684,1.846)	0.644	1.150 (0.698,1.894)	0.584
IQR3	157	1.920 (1.207,3.053)	**0.006**	1.728 (1.075,2.779)	**0.024**	1.720 (1.067,2.774)	**0.026**	1.724 (1.007,2.953)	**0.047**	1.805 (1.041,3.130)	**0.036**
IQR4	151	1.386 (0.860,2.234)	0.180	1.156 (0.705,1.895)	0.567	1.145 (0.696,1.884)	0.593	1.150 (0.558,2.370)	0.705	1.319 (0.594,2.927)	0.497
Iron (mg)											
IQR1	170	Ref.		Ref.		Ref.		Ref.		Ref.	
IQR2	162	1.477 (0.918,2.376)	0.108	1.198 (0.732,1.961)	0.471	1.171 (0.714,1.921)	0.532	1.177 (0.710,1.952)	0.527	1.204 (0.725,2.002)	0.473
IQR3	158	2.011 (1.257,3.216)	**0.004**	1.717 (1.062,2.778)	**0.028**	1.661 (1.024,2.695)	**0.040**	1.680 (0.983,2.870)	0.058	1.768 (1.023,3.053)	**0.041**
IQR4	158	1.577 (0.980,2.539)	0.061	1.191 (0.724,1.961)	0.491	1.188 (0.721,1.959)	0.499	1.215 (0.626,2.355)	0.565	1.382 (0.677,2.825)	0.374
Manganese (mg)											
IQR1	158	Ref.		Ref.		Ref.		Ref.		Ref.	
IQR2	179	1.389 (0.859,2.246)	0.180	1.280 (0.783,2.091)	0.325	1.284 (0.785,2.099)	0.320	1.292 (0.784,2.131)	0.315	1.334 (0.806,2.206)	0.262
IQR3	153	2.315 (1.428,3.751)	**0.001**	1.985 (1.210,3.257)	**0.007**	1.906 (1.157,3.138)	**0.011**	1.936 (1.127,3.324)	**0.017**	2.080 (1.193,3.628)	**0.010**
IQR4	158	1.629 (1.000,2.654)	0.050	1.280 (0.771,2.126)	0.340	1.278 (0.768,2.125)	0.345	1.320 (0.678,2.567)	0.414	1.549 (0.752,3.193)	0.235

Note**: Model 1,** Crude model. **Model 2,** Adjusted for age. **Model 3,** Adjusted for age, educational level. **Model 4,** Adjusted for age, educational level, total food intake per day (g). **Model 5,** Adjusted for age, educational level, total food intake per day (g), Total energy intake per day (KJ). ***Subclassification (based on IQR):*** **subtype 1**, 0 ≤ element quantification ≤ 0.25; **subtype 2**, 0.25 < element quantification ≤ 0.50; **subtype 3**, 0.50 < element quantification ≤ 0.75; **subtype 4**, 0.75 < element quantification ≤ 1. ***Abbreviation*****:** PLS, prostatitis-like symptoms; No, number; y, years, BMI, body mass index; kg, kilogram; m, meter; KJ, kilojoule; mg, milligram; ug, microgram; IQR, interquartile range.

Supplementary Table S4. Multiple logistic regression models were constructed by adding confounding factors gradually to identify high-risk elements intake associated with NIANS in daily diet.

Variables	No.	Crude model OR (95% CI)	P-value	Adjusted model OR (95% CI)	P-value
Moisture (g)					
1	261	Ref.		Ref.	
2	264	1.069 (0.758,1.508)	0.703	1.054 (0.746,1.490)	0.766
3	260	1.530 (1.076,2.176)	**0.018**	1.554 (1.090,2.214)	**0.015**
4	255	1.197 (0.845,1.697)	0.312	1.207 (0.850,1.714)	0.294
Protein (g)					
1	269	Ref.		Ref.	
2	265	1.382 (0.981,1.948)	0.064	1.388 (0.983,1.960)	0.062
3	246	1.345 (0.949,1.908)	0.096	1.353 (0.952,1.922)	0.092
4	260	1.548 (1.094,2.191)	**0.014**	1.535 (1.083,2.175)	**0.016**
Fat (g)					
1	274	Ref.		Ref.	
2	255	1.764 (1.246,2.497)	**0.001**	1.794 (1.263,2.547)	**0.001**
3	249	1.56 (1.102,2.207)	**0.012**	1.549 (1.092,2.196)	**0.014**
4	262	1.668 (1.183,2.352)	**0.004**	1.671 (1.183,2.362)	**0.004**
Carbohydrate (g)					
1	270	Ref.		Ref.	
2	264	1.006 (0.714,1.416)	0.973	0.994 (0.704,1.402)	0.971
3	251	1.253 (0.883,1.779)	0.206	1.269 (0.892,1.806)	0.186
4	255	1.146 (0.810,1.621)	0.443	1.145 (0.808,1.623)	0.446
Dietary fiber (g)					
1	279	Ref.		Ref.	
2	264	1.086 (0.773,1.525)	0.635	1.129 (0.802,1.592)	0.487
3	243	1.204 (0.850,1.707)	0.297	1.227 (0.864,1.743)	0.253
4	254	1.253 (0.887,1.769)	0.201	1.266 (0.895,1.792)	0.182
Cholesterol (mg)					
1	267	Ref.		Ref.	
2	266	0.993 (0.706,1.398)	0.969	0.975 (0.691,1.374)	0.883
3	257	1.127 (0.797,1.593)	0.498	1.135 (0.801,1.607)	0.477
4	250	1.506 (1.056,2.148)	**0.024**	1.516 (1.061,2.166)	**0.022**
Ash (g)					

1	272	Ref.		Ref.	
2	265	1.233 (0.877,1.734)	0.228	1.239 (0.879,1.747)	0.221
3	248	1.560 (1.098,2.218)	**0.013**	1.591 (1.116,2.267)	0.100
4	255	1.312 (0.929,1.853)	0.124	1.302 (0.920,1.842)	0.136
Vitamin A (ug)					
1	273	Ref.		Ref.	
2	248	1.293 (0.912,1.832)	0.149	1.311 (0.923,1.861)	0.130
3	264	1.201 (0.853,1.690)	0.293	1.191 (0.845,1.679)	0.318
4	255	1.309 (0.926,1.851)	0.127	1.315 (0.929,1.862)	0.123
Carotene (ug)					
1	275	Ref.		Ref.	
2	257	1.289 (0.913,1.821)	0.149	1.354 (0.956,1.919)	0.088
3	253	1.299 (0.918,1.838)	0.140	1.335 (0.941,1.894)	0.105
4	255	1.082 (0.768,1.526)	0.651	1.113 (0.788,1.573)	0.542
Retinol (ug)					
1	256	Ref.		Ref.	
2	255	1.074 (0.758,1.522)	0.687	1.090 (0.768,1.547)	0.630
3	259	1.274 (0.899,1.806)	0.174	1.295 (0.912,1.840)	0.148
4	270	1.626 (1.145,2.310)	**0.007**	1.649 (1.158,2.348)	**0.006**
Thiamin (mg)					
1	269	Ref.		Ref.	
2	255	1.274 (0.903,1.800)	0.168	1.285 (0.908,1.818)	0.157
3	256	1.790 (1.258,2.546)	**0.001**	1.788 (1.255,2.549)	**0.001**
4	260	1.276 (0.906,1.799)	0.164	1.276 (0.903,1.803)	0.166
Riboflavin (mg)					
1	265	Ref.		Ref.	
2	260	1.468 (1.038,2.075)	**0.030**	1.483 (1.047,2.100)	**0.027**
3	256	1.478 (1.044,2.092)	**0.028**	1.502 (1.059,2.132)	**0.023**
4	259	1.582 (1.117,2.240)	**0.010**	1.600 (1.128,2.270)	**0.008**
Vitamin C (mg)					
1	271	Ref.		Ref.	
2	263	1.316 (0.931,1.861)	0.119	1.308 (0.923,1.853)	0.131
3	259	1.060 (0.752,1.494)	0.741	1.046 (0.741,1.478)	0.798
4	247	1.130 (0.797,1.601)	0.493	1.140 (0.803,1.619)	0.463
Vitamin E [total (mg)]					
1	274	Ref.		Ref.	
2	262	1.293 (0.918,1.823)	0.142	1.331 (0.942,1.882)	0.105

3	257	1.252 (0.888,1.767)	0.200	1.284 (0.908,1.816)	0.158
4	247	1.294 (0.914,1.834)	0.147	1.291 (0.910,1.832)	0.153
Vitmain E-α (mg)					
1	276	Ref.		Ref.	
2	253	1.221 (0.865,1.724)	0.256	1.256 (0.888,1.777)	0.198
3	255	1.365 (0.966,1.930)	0.078	1.369 (0.966,1.938)	0.077
4	256	1.266 (0.897,1.786)	0.179	1.258 (0.890,1.777)	0.194
Vitamin E-(β+γ) (mg)					
1	282	Ref.		Ref.	
2	259	0.860 (0.612,1.208)	0.384	0.885 (0.628,1.248)	0.487
3	250	1.097 (0.776,1.552)	0.599	1.102 (0.777,1.564)	0.584
4	249	1.127 (0.796,1.596)	0.499	1.121 (0.791,1.589)	0.521
Vitmain E-δ (mg)					
1	269	Ref.		Ref.	
2	262	0.898 (0.636,1.268)	0.542	0.894 (0.632,1.265)	0.528
3	259	0.840 (0.595,1.186)	0.322	0.846 (0.597,1.197)	0.345
4	250	1.131 (0.794,1.610)	0.496	1.124 (0.788,1.603)	0.519
Calcium (mg)					
1	262	Ref.		Ref.	
2	254	1.005 (0.711,1.422)	0.976	1.018 (0.719,1.442)	0.919
3	257	1.465 (1.031,2.082)	**0.033**	1.490 (1.047,2.123)	**0.027**
4	267	1.367 (0.967,1.933)	0.077	1.366 (0.964,1.936)	0.079
Zinc (mg)					
1	270	Ref.		Ref.	
2	263	1.161 (0.825,1.633)	0.392	1.176 (0.834,1.659)	0.354
3	245	1.503 (1.056,2.138)	**0.023**	1.522 (1.067,2.170)	**0.020**
4	262	1.439 (1.019,2.032)	**0.039**	1.448 (1.023,2.049)	**0.037**
Magnesium (mg)					
1	274	Ref.		Ref.	
2	262	1.480 (1.050,2.085)	**0.025**	1.498 (1.060,2.119)	**0.022**
3	257	1.530 (1.083,2.162)	**0.016**	1.529 (1.080,2.165)	**0.017**
4	247	1.410 (0.996,1.996)	0.053	1.415 (0.997,2.007)	0.052
Selenium (mg)					
1	273	Ref.		Ref.	
2	257	1.095 (0.777,1.543)	0.605	1.090 (0.771,1.541)	0.627
3	257	1.396 (0.986,1.975)	0.060	1.396 (0.983,1.980)	0.062
4	253	1.252 (0.886,1.771)	0.203	1.265 (0.893,1.793)	0.186

Phosphorus (mg)					
1	268	Ref.		Ref.	
2	260	1.102 (0.782,1.553)	0.578	1.122 (0.794,1.585)	0.516
3	249	1.492 (1.049,2.121)	**0.026**	1.504 (1.055,2.142)	**0.024**
4	263	1.425 (1.008,2.013)	**0.045**	1.422 (1.004,2.014)	**0.048**
Potassium (mg)					
1	274	Ref.		Ref.	
2	257	1.503 (1.064,2.122)	**0.021**	1.534 (1.083,2.172)	**0.016**
3	250	1.562 (1.102,2.212)	**0.012**	1.601 (1.127,2.274)	**0.009**
4	259	1.450 (1.028,2.044)	**0.034**	1.459 (1.033,2.062)	**0.032**
Sodium (mg)					
1	280	Ref.		Ref.	
2	263	1.542 (1.095,2.172)	**0.013**	1.554 (1.100,2.195)	**0.012**
3	243	1.401 (0.990,1.984)	0.057	1.438 (1.012,2.042)	**0.042**
4	254	1.410 (1.000,1.989)	0.050	1.409 (0.997,1.991)	0.052
Iron (mg)					
1	271	Ref.		Ref.	
2	260	1.241 (0.881,1.749)	0.217	1.252 (0.886,1.767)	0.203
3	250	1.464 (1.032,2.077)	**0.033**	1.472 (1.035,2.094)	**0.031**
4	259	1.336 (0.946,1.886)	0.100	1.338 (0.946,1.892)	0.100
Copper (mg)					
1	279	Ref.		Ref.	
2	265	1.270 (0.904,1.784)	0.168	1.297 (0.921,1.828)	0.137
3	251	1.176 (0.834,1.659)	0.354	1.159 (0.820,1.637)	0.404
4	245	1.481 (1.043,2.103)	**0.028**	1.503 (1.057,2.139)	**0.023**
Manganese (mg)					
1	275	Ref.		Ref.	
2	258	0.837 (0.595,1.178)	0.308	0.845 (0.599,1.191)	0.337
3	248	1.412 (0.991,2.010)	0.056	1.406 (0.985,2.006)	0.061
4	259	1.155 (0.818,1.631)	0.412	1.153 (0.815,1.629)	0.421

Note: Adjusted for economic pressure. ***Subclassification (based on IQR):*** **subtype 1**, 0 ≤ element quantification ≤ 0.25; **subtype 2**, 0.25 < element quantification ≤ 0.50; **subtype 3**, 0.50 < element quantification ≤ 0.75; **subtype 4**, 0.75 < element quantification ≤ 1. ***Abbreviation***: NIANS, noninflammatory-abnormal symptoms; Ref., reference; No., number; y, years, BMI, body mass index; kg, kilogram; m, meter; KJ, kilojoule; mg, milligram; ug, microgram; IQR, interquartile range.
